# A Modelization of the Propagation of COVID-19 in Regions of Spain and Italy with Evaluation of the Transmission Rates Related to the Intervention Measures

**DOI:** 10.3390/biology10020121

**Published:** 2021-02-05

**Authors:** Raul Nistal, Manuel de la Sen, Jon Gabirondo, Santiago Alonso-Quesada, Aitor J. Garrido, Izaskun Garrido

**Affiliations:** 1 Department of Electricity and Electronics, University of the Basque Country UPV/EHU, 48940 Leioa, Spain; manuel.delasen@ehu.eus (M.d.l.S.); Jgabirondo97@gmail.com (J.G.); santiago.alonso@ehu.eus (S.A.-Q.); 2 Automatic Control Group (ACG), Department of Automatic Control and Systems Engineering, Institute of Research and Development of Processes (IIDP), University of the Basque Country UPV/EHU, 48013 Bilbao, Spain; aitor.garrido@ehu.eus (A.J.G.); izaskun.garrido@ehu.eus (I.G.)

**Keywords:** infectious disease, epidemiology, modelization, digital health, COVID-19, global health

## Abstract

**Simple Summary:**

Two different mathematical models are proposed in order to describe the spreading of COVID-19 through the different provinces and regions of Spain and Italy. The models will divide the populations of both countries in three categories: the subpopulation susceptible to be infected of the disease, the subpopulation which is already infected and thus is infectious, and the subpopulation which has already recovered from the disease so is considered immune. The transmission rate is calculated within these models while considering the different locations and, more importantly, the lockdown measures implemented during 2020. The efficiency of these measures is compared between the areas of infection and the different levels of lockdown.

**Abstract:**

Two discrete mathematical SIR models (Susceptible-Infectious-Recovered) are proposed for modelling the propagation of the SARS-CoV-2 (COVID-19) through Spain and Italy. One of the proposed models is delay-free while the other one considers a delay in the propagation of the infection. The objective is to estimate the transmission, also known as infectivity rate, through time taking into account the infection evolution data supplied by the official health care systems in both countries. Such a parameter is estimated through time at different regional levels and it is seen to be strongly dependent on the intervention measures such as the total (except essential activities) or partial levels of lockdown. Typically, the infectivity rate evolves towards a minimum value under total lockdown and it increases again when the confinement measures are partially or totally removed.

## 1. Introduction

The advance of the disease caused by the corona virus SARS-CoV-2 (COVID-19) surprised world population in the early 2020 as its rapid spread and virulence affected the lives of millions of people and caused thousands of deaths. Given the importance of this situation, a great number of mathematical models explaining the spread of the disease have been proposed [1,2,3], with a set of prescribed characteristic parameters defining the infection over time in a given population. While there is a great number of different models we can use to describe a disease like this [4,5,6], due to the limited data at the time of writing this paper, we have chosen a SIR model, which fits better the available data. Another reason to use the SIR model, is that it has shown a great range of versatility for many types of epidemiological studies [7,8,9,10,11,12]. Usually, the network of interactions that define the spread of an infectious disease is modelled with differential equations. It involves different types of infected subpopulations, or susceptible to be infected [13,14,15], as well as the transitions between them. These transitions and the dynamics that derives from them depend on the type of disease and the circumstances in which the infection occurs. Unfortunately, the countries suffering from COVID-19 are not coherently documenting the state of the disease: the data regarding the impact of the epidemic on the society has shown a great variation depending on the different methods of diagnostics, treatment and surveillance of the affected population [16]. Even the politics may play a part in the transparency of the provided data [17,18,19]. This paradoxical situation of the data acquisition, plus the inherent difficulties for designing a proper mathematical model, is specially relevant when there is a need to define the infectivity rate. The infectivity rate (β), can be explained by defining first the force of infection λ, which is the rate at which susceptible individuals contract the infection per capita. This way, the rate at which new infected appear is λS, being S the value of susceptible subpopulation. This force of infection is proportional to the number of infectious individuals so we define the transmission rate β as λ=βI, which will depend on the average number of contacts an individual encounters during certain time and the probability of transmission of the disease in a contact between an infectious individual and a susceptible one [14]. This is the parameter we can infer from the dynamics of the infected subpopulation. Although traditionally the biggest determinator of the virality of a disease is the Reproduction number [14], we have chosen to study the infectivity rate due to its direct relation to the parameters involved in the strategies for controlling the disease [20,21]. The time scale of the data presented in this paper is more adequate with the rapid changing values of the infectivity rate rather than a more traditional not so fluctuating reproduction number. By selecting different countries with similar economy, internal politics, health care system, population density and age and social interactions, the possible interference related to these factors, that may affect the spreading of the disease in a population, are reduced to the minimum. Thus, we have chosen Spain and Italy as they share many of these characteristics rather adequately [22,23,24,25]. Two different discrete SIR models describing the different regions of Spain and Italy at different organization entity levels (Provinces, Regions or Autonomous Communities and Countries as a whole) will be proposed, taking into account the different stages in which they have experienced the spread of the disease: From the principal one to the following de-escalating lockout stages, or phases. While it is possible that different strains of the virus are spreading at the same time, the general assumption will be that others aspects of the disease, such as the average time of incubation of the virus and the average recovery of an average infectious individual is the same through all of the time measured. Then, the only parameter which is really available to control and comparable between different healthcare strategies and social distancing will be the infectivity rate and it will be calculated in their different lock-down stages given these two models. This paper is structured as follows: In the first two sections, we have done this small introduction and present the SIR models which we will use during our analysis. In Section 3 we will explain the methods for obtaining the infectivity rate and the rest of parameters from the given data. Finally, the results and conclusions derived from them will be presented in the Section 4 and Section 5.

## 2. The Models

**Figure 1:**
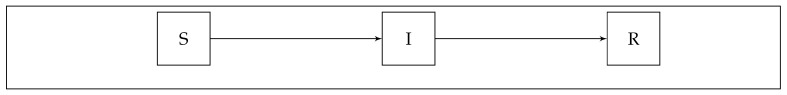
Scheme of the SIR Model.

Two different SIR models will be proposed in this section. In these models, the total population is divided into three different subpopulations: susceptible, infectious and removed subpopulation, as seen in the following transition scheme (Figure 1):

The susceptible and the infectious or infected subpopulation, as their names suggest, correspond respectively to the individuals that are susceptible to be infected and the individuals already infected. The removed subpopulation, also known sometimes as recovered, corresponds to the non-susceptible and non-infected individuals, because they acquire immunity or die. The transition rates governing interactions between the three subpopulations will be described thoroughly by discrete equations representing the proportion of individuals in each subpopulation in one day. The values of the recovery rates (transition from I to R) will be obtained by taking into account medical records from the data acquired from early medical cases [26,27,28], while the infectivity rates (transition from S to I) parameters of these models will be later adjusted to the real data.

### 2.1. Non-Delayed Model

A SIR traditional discrete model, in which the daily growth of infectious is directly proportional to the value of the susceptible and infectious subpopulation at the time of the measurement, is first proposed. This basic model will be compared to more complex ones that will be presented later. The equations of the dynamics between subpopulations would be:

(1) $$\begin{array}{ccc} S_{i+1} & = & S_{i}-\beta \left(S_{i}I_{i}\right) \end{array}$$

(2) $$\begin{array}{ccc} I_{i+1} & = & I_{i}+\beta \left(S_{i}I_{i}\right)-\gamma I_{i} \end{array}$$

(3) $$\begin{array}{ccc} R_{i+1} & = & R_{i}+\gamma I_{i} \end{array}$$

with *S*, *I* and *R* the values of the proportion of the susceptible, infectious and removed subpopulation respectively. The values for the number of individuals in each of the subpopulations are measured daily, so the subscripts of *S*, *I* and *R* correspond to the day i+1 and *i* respectively. β corresponds to the infectivity rate and γ to the average rate of recovery from being infected, or recovery rate. These models will assume that the recovered subpopulation includes the dead caused by the disease, as these individuals will affect the dynamics of the disease in the same way as they would do if they just be recovered and immune. A main advantage of this simple model is that, in order to estimate the infectivity rate β of a susceptible individual, it is not necessary to know the exact value of the recovery rate γ. Additionally, the rate of change of the subpopulations in this model is applied in such small periods of time, corresponding to the different stages of the lockdown. The rate of new individuals due to immigration or newborns is dismissed, as well as the mortality of the healthy individuals.

### 2.2. Delayed Model

A SIR model with delays, in which the daily growth of infectious subpopulation is proportional to the value of the susceptible and infectious subpopulations during several previous days is now proposed, where the incubation and the recovery times obtained from medical records [29,30] are taken into account. The equations of the dynamics would be a variation of those of the previous section. Namely:

(4) $$\begin{array}{ccc} S_{i+1} & = & S_{i}-\sum_{k=k_{0}}^{k_{1}}\beta_{k}\left(S_{i-k}I_{i-k}\right) \end{array}$$

(5) $$\begin{array}{ccc} I_{i+1} & = & I_{i}+\sum_{k=k_{0}}^{k_{1}}\beta_{k}\left(S_{i-k}I_{i-k}\right)-\sum_{l=l_{0}}^{l_{1}}\gamma_{l}I_{i-l} \end{array}$$

(6) $$\begin{array}{ccc} R_{i+1} & = & R_{i}+\sum_{l=l_{0}}^{l_{1}}\gamma_{l}I_{i-l} \end{array}$$

with $\beta_{k}$ and $\gamma_{l}$ the infectivity and recovery rates respectively, with different values depending on the probability of transition at different k,l days previous to the *i* current day, since day $k_{0},l_{0}$ to day $k_{1},l_{1}$ respectively. As in the previous model, the values the subscripts of S, I and R correspond to the day i+1, *i*, i−k and i−l for integers $k\in [k_{0},k_{1}]$ and $l\in [l_{0},l_{1}]$ respectively. Observe that in this model, although an exposed subpopulation is not explicitly described, there is implicitly a delay between the moment of contagion and the moment when the susceptible individual becomes infectious. As in the SEIR models, we can consider the influence of the exposed subpopulation although the data regarding these individuals are not explicit. The main difference would be that in this situation the exposed individuals are not affecting the dynamics in any way different than the susceptible subpopulation.

## 3. Estimation of the Infectivity Rate β

An estimation of the value of the infectivity parameter will be made through linear regression using the data provided by the health authorities of Spain and Italy. We will take into account the fact that Melilla and Ceuta are special autonomic cities in Spain, with little population and a very special dynamics so they will be excluded and the total number of provinces in Spain will be set to 50. Although the territorial divisions in Italy and Spain are different, with 20 and 17 regions/autonomous communities and 107 and 50 provinces respectively, more than 75% of the regions/autonomous communities are in the range of 500–1500 k inhabitants and 75% of the provinces of both countries are in the range of 200–1000 k inhabitants, and both of them present a density and population numbers quite similar compared to other countries in Europe. We will study the effect of the lockdown and the prophylactic measures, such as the different levels of social distancing and the probability of infection, on the infectivity rate parameter β. The actual data provided from official sources cannot always be used directly to study this parameter as the novelty of the situation does not provide a standardized method for discharging a patient and/or find a reliable exact number of infectious individuals at any time. We will take the values of the accumulated total cases and the daily new infections and new recovered ones, which are the easiest data to find in the official repositories, from the governments of Spain and Italy [31,32]. Covid-19 data are available from 24 February for Italy, and the data available for Spain is from 1 January, but we have decided that the first analysis period will be from 18 February to 3 May, when a considerable rise is observed in new daily cases. The analysis in both countries ends at 13 September. The total cases will include the infected, recovered and death individuals, and the current susceptible ones will be all that are left from the total population, which will be 1 as it is normalized. Then, the value of the susceptible subpopulation at any day *i* would be $S_{i}=\left(1-CumulativeCases_{i}\right)$. Thus, after combining Equations (2) and (3), we get that

(7) $$\begin{array}{c} \left(I_{i+1}-I_{i}\right)+\left(R_{i+1}-R_{i}\right)=\Delta I_{i}+\Delta R_{i}=\Delta {\left(CumulativeCases\right)}_{i}=\beta \left(S_{i}I_{i}\right) \end{array}$$

From here we will get the equation of β, for each data point, corresponding to a day:

(8) $$\beta =\frac{\Delta {\left(CumulativeCases\right)}_{i}}{I_{i}*\left(1-CumulativeCases_{i}\right)}$$

This value will obviously be affected by multiple factors, such as the existence of local super spreader nodes or different weather conditions [33,34] which we are unable either to control or observe with such limited data. However, the main factor that influences the value of β will be the average contacts per day of a susceptible individual, and the probability of an infection as a result of a contact with an infected individual [26]. This influence can be properly compartmentalized in the intervals of time in which the population have shown different social interactions as the stages of their respective lockdown measures have been implemented [35,36] as follows:

- In Italy, the lockdown began on 8 March in Lombardia and 14 provinces while on 10 March in the rest of the country, and it ended at 4 May.
- The lockdown in Spain took place from 15 March to 4 May.
- On 23 March there was a tightening of the measures, but we considered that the habits and the social contact were not altered enough to add another phase.
- Italy has a criteria for establishing the dates of de-escalating stages following the lockdown determined nationally, so in 18 May the whole country was in the phase 2, on 25 May in the phase 3, on 3 June in the phase 4 and on 15 June the normality was reached. However, in Spain it was independently chosen at each Autonomous Community until 21 June when the final phase ended in all the country.

In the case of the delayed SIR model, the values of $\beta_{k}$ will describe the probability of an individuals to present some delay time from the moment of infection to developing the disease. A bell-shaped curve symmetrically distributed with no skew around the average value for the delay is set, with values tapering off as they go further away from the maximum central value of a typical incubation time. We make so we can take into account the individuals who present symptoms at average incubation time as well as the deviation of this value, which would be around 4 days [29,30] Thus, the infectivity rates will be defined as $\beta_{k}=\beta_{0}a_{k}$ with $a_{k}$ a Gaussian distribution such that

(9) $$a_{k}=ae^{-\frac{{\left(k-\mu \right)}^{2}}{2\sigma^{2}}},\;\mathrm{and}\;\sum_{k_{0}}^{k_{1}}a_{k}=1,\;\mathrm{with}\;a=\frac{1}{\sum_{k_{0}}^{k_{1}}a_{k}}$$

Then the equation of β for the delayed SIR model will be:

(10) $$\beta_{0}=\frac{\Delta {\left(CumulativeCases\right)}_{i}}{\sum_{k_{0}}^{k_{1}}a_{k}I_{i-k}*\left(1-CumulativeCases_{i-k}\right)}$$

The transmission rate β is then defined so that, multiplied by the number of infectious and the susceptible individuals from the previous day or days, gives the value of the new cases. The value of β is obtained by taking the method of least squares, or linear regression with a null intercept.

(11) $$Y∼\beta X+\delta ⟺\left\{\begin{array}{c} Y=\Delta {\left(CumulativeCases\right)}_{k+1}\\ X=S_{k}I_{k}/\sum_{k_{0}}^{k_{1}}a_{k}I_{i-k}S_{i-k}\\ \delta =0 \end{array}\right.\Delta {\left(CumulativeCases\right)}_{k+1}=\beta S_{k}I_{k}/\sum_{k_{0}}^{k_{1}}a_{k}I_{i-k}S_{i-k}$$

The infectivity rate β will be then calculated in two ways by linear regression assuming the intercept is null: First, the infectious rate β will be calculated with the data available in each phase of the social distancing measures adopted by the territorial government. The other one, the continuous β will be calculated each day using the 15-day periods previous to this day. In this way, a continuous β will be calculated with the data obtained from the first 15 days and it will assigned to the 15th day. Then, the period will be set from the day 2 to 16 and the outcome assigned to the 16th day, and so on. In this way, a graphical representation of the infectivity rate is obtained as if it were a continuous parameter from the day 15 to the last day of the data, which may give more insight of the fluctuations of the infectivity rate in each phase, as well as it show the changes of the lockdown strategies right away. In the pre-lockdown stage of Italy, where there is a lack of sufficient data points, a 7-day period β will be established as a special case in order to estimate the value of the transmission rate in this moments.

## 4. Results

In this section we will show the parameter β adjusted by linear regression for the different regions of Spain and Italy based on the SIR models which derive the Equations (8) and (10) respectively. The data for the spreading of Covid-19 disease are available from 24 February for Italy, so the first analysis period of 15 days ends on 9 March when the first calculated infectivity rate is assigned. The data available for Spain is from 1 January, and we have decided that the first analysis period will be from 18 February to 3 May, when a considerable rise is observed in new daily cases. The analysis in both countries ends on 13 September. Additionally, another value of β is daily calculated as the average value for the last previous 15 days. In every case, the curves describing the cumulative and direct values of the infected individuals are smoothed through weighted moving average [37], in order to filter out the possible noises in measurements and bureaucratic errors when publishing the results. The range of the graphs are limited, and out of range values will be considered an outliers. Those values may appear at the beginning of the pandemic when the new cases grow fast and also along summer, when the low number of infectious individuals causes that a little number of new cases increases the rate of infection.

### 4.1. Non-Delayed SIR Model

**Figure 2:**
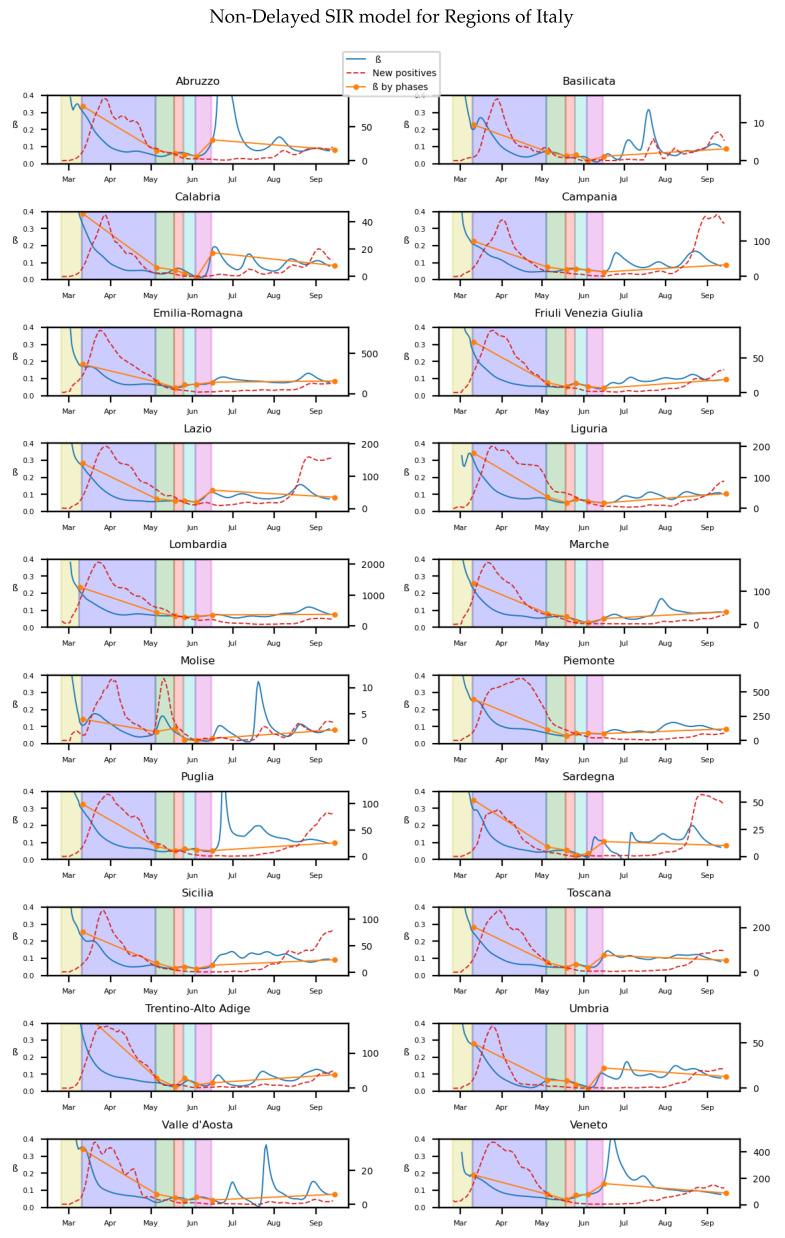
The y axis on the left on each subfigure correspond to the parameter β, while the one on the right to the number of new registered infectious. Each background color represents a different stage of lockdown of Italy: Lockdown, phase 1,2,3,4. White background correspond to pre-lockdown and normality. Red dotted line: The value of new infected individuals at that day. Blue thin line: A 15-days average of β at that day calculated from Equation (8). Orange thick line: A linear regression of β during each stage.

For the non-delayed SIR model, we get Figure 2 from the data obtained from the Italian ministry of health [38].

**Figure 3:**
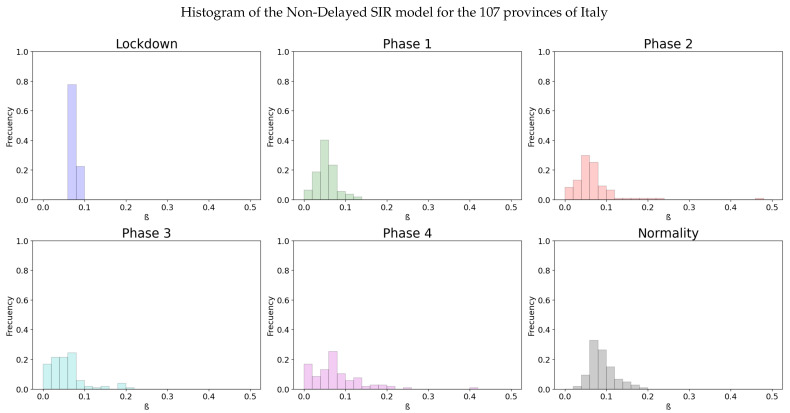
A distribution of the values for β for the different stages of lockdown in the different provinces of Italy.

We can see the different continuous and average infectivity rates calculated for each of the 20 regions of Italy depending on the lockdown stage at which they are in time. A more clear visualization of this fact is presented in Figure 3. Here the particular value of β for the more than 100 provinces of Italy are also calculated through linear regression for the diverse stages of the lockdown. Then, an histogram for the distribution of the values is made. We can see it more easily at Figure 3 that the lockdown reduces the infectivity rate for most of the provinces, compared to the normal final state at September.

The statistics from Figure 3 are displayed in the following Table 1.

**Figure 4:**
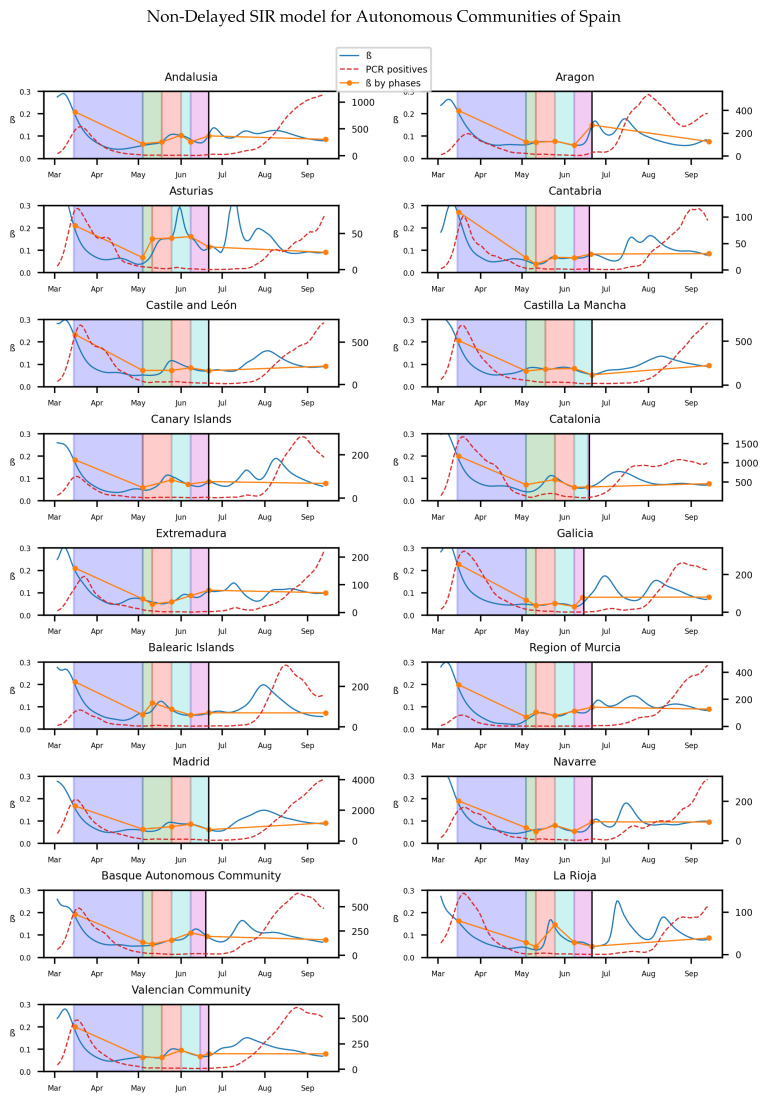
The y axis on the left on each subfigure correspond to β, while the one on the right to the number of new infected. Each background color represents a different stage of lockdown of Spain: Lockdown, phase 0,1,2,3. White background correspond to pre-lockdown and normality. Red dotted line: The value of new infected individuals at that day. Blue thin line: A 15-days average of β at that day calculated from Equation (8). Orange thick line: A linear regression of β during each stage.

Statistically significant differences are observed in the histograms through the use of Z test [39], as the size of the samples is large enough. The change in transmission rate has changed significantly between two stages when the *p*-value of the test is low enough. In the corresponding tables for each histogram, the *p*-value indicates the result for the Z test between each stage and the previous one. We can see the influence of the high infectivity rate after the lockdown affecting to all provinces if we look at the mean value of β. The effects of the strict isolation measures are eventually reflected in the mean value of β at phase 1. Moreover, the variance of the infectivity rates obtained from different provinces of Italy shows that the lockdown induces an homogeneous effect in the populations. The histogram Figure 3 and Table 1 show that there are significant differences in the value of the infectivity rate at the different phases and normality. In Spain, the same method is used to get the continuous and average infectivity rates for the 17 Autonomous Communities, which are shown in the Figure 4.

Again, as in Italy, we will show the distribution of the infectivity rates of the different provinces of Spain (50) in an histogram for each stage of the lockdown.

**Figure 5:**
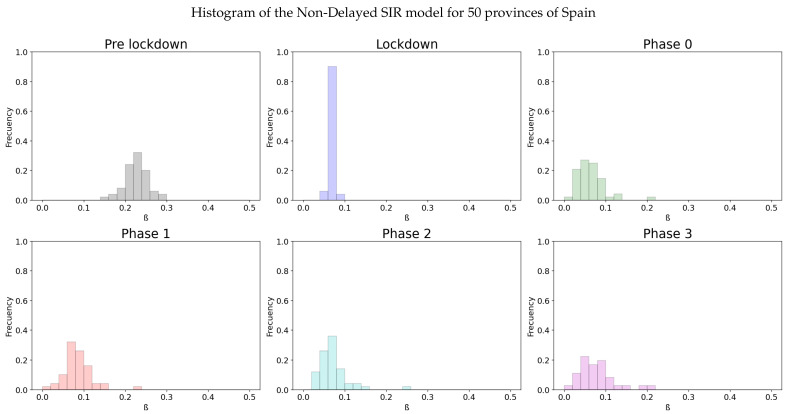
A distribution of the values for β for the different stages of lockdown in the different provinces of Spain.

We can see the results in Table 2 and Figure 5.

Additionally, we can observe the homogeneity of the infectivity rate from the lockdown. The decrease of β during lockdown is clear, as it can be seen in the mean values and confidence intervals from pre-lockdown and lockdown period. The contrast of the values of β when the measures are even more relaxed in the following phases is not so well appreciated.

### 4.2. Delayed SIR Model

**Figure 6:**
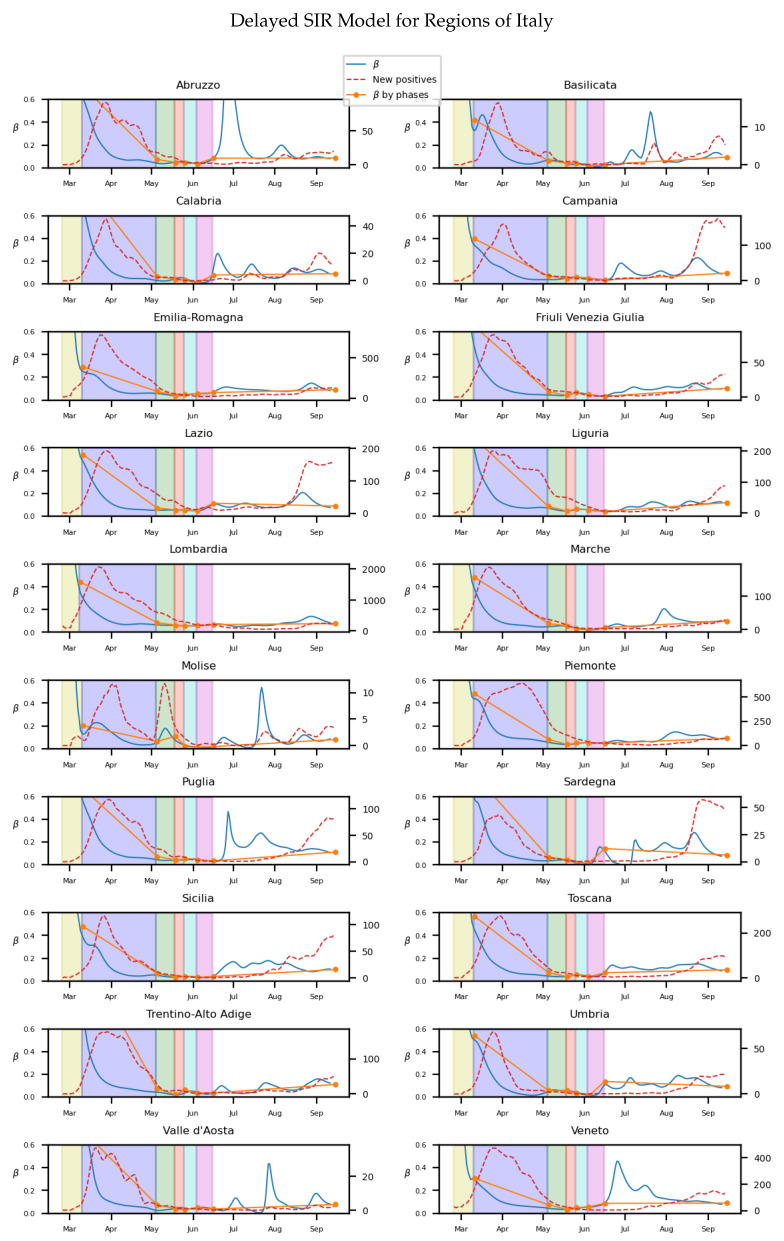
The y axis on the left on each subfigure correspond to the parameter β, while the one on the right to the number of new registered infectious. Each background color represents a different stage of lockdown of Italy: Lockdown, phase 1,2,3,4. White background correspond to pre-lockdown and normality. Red dotted line: The value of new infected individuals at that day. Blue thin line: A 15-days average of β at that day calculated from Equation (10). Orange thick line: A linear regression of β during each stage.

While we process the same data for the delayed SIR model as in the previous section, we will set additional parameters for the Gaussian distribution describing the delay. From defining $a_{k}$ in Equation (9), we consider k∈[1,6], μ=4 and σ=2, so the significant $\beta_{k}$, corresponding to the approximate interquartile range of the incubation period will be around 2–6 days, with the previous 4th day presenting the maximum value [40]. We obtain after processing the data the Figure 6. The results, while similar to the previous model, present infectivity rates which are more softened and homogeneous.

**Figure 7:**
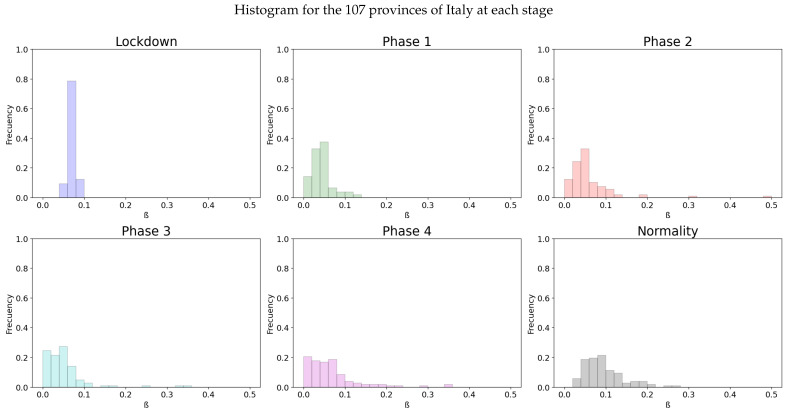
A distribution of the values for β for the different stages of lockdown in the different provinces of Italy.

As in the previous model, the individual values of the 107 provinces of Italy are also calculated through linear regression for the diverse stages of the lockdown. An histogram for the distribution of the values at Figure 7 and their statistics presented in Table 3 show the different values of β.

**Figure 8:**
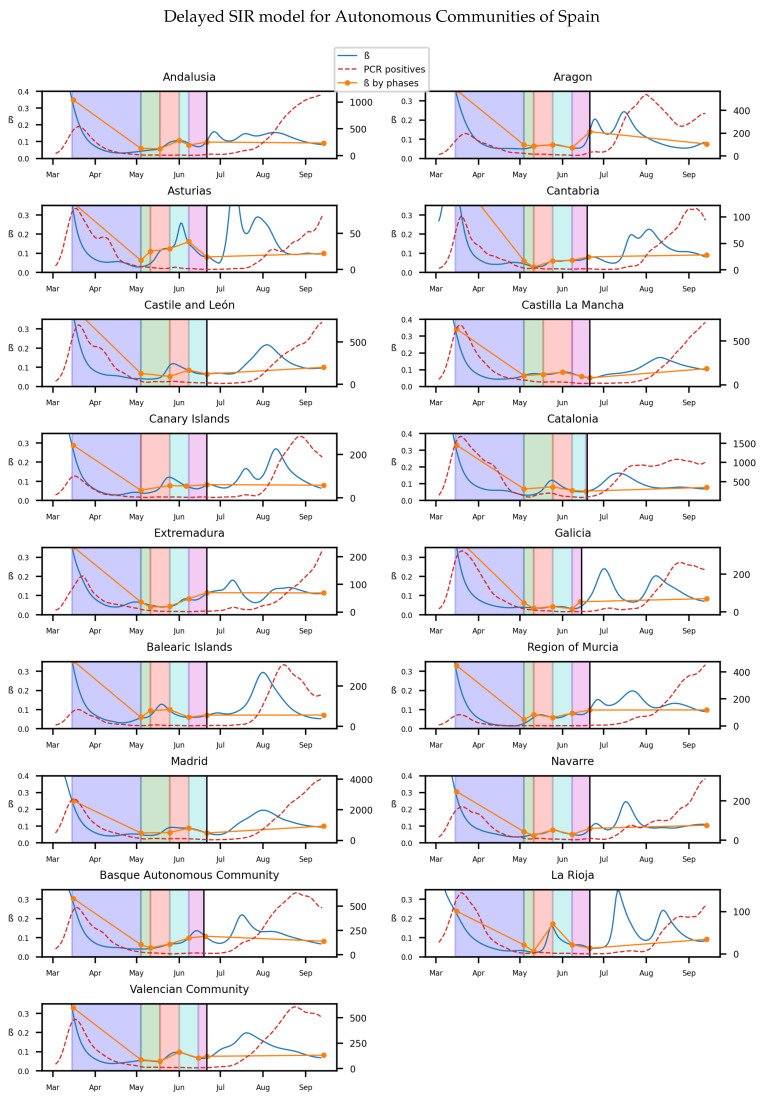
The y axis on the left on each subfigure correspond to β, while the one on the right to the number of new infected. Each background color represents a different stage of lockdown of Spain: Lockdown, phase 0,1,2,3. White background correspond to pre-lockdown and normality. Red dotted line: The value of new infected individuals at that day. Blue thin line: A 15-days average of β at that day calculated from Equation (10). Orange thick line: A linear regression of β during each stage.

The homogeneity of the values of β at the diverse regions of Italy in the lockdown is maintained as it is in previous model (seen in Table 1). Also, the effect of social isolation can be seen in phase 1, and there is significant difference between the infectivity rate from the phase 1 and normality. For Spain we will get, as in Italy and the previous model, the Figure 8 for the infectivity rate β at each stage of lockdown and each region

**Figure 9:**
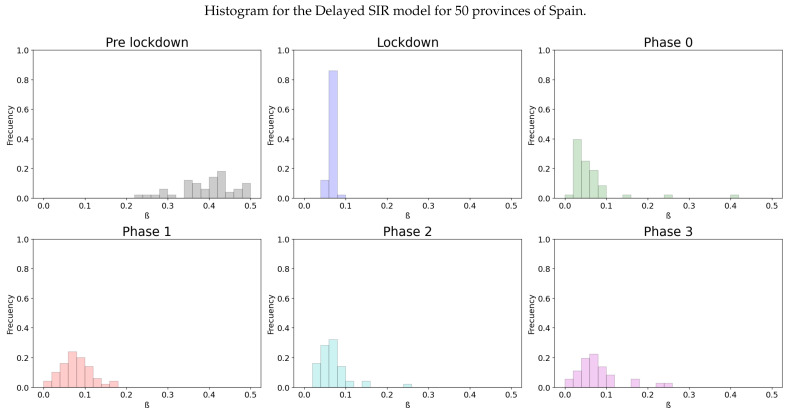
A distribution of the values for β for the different stages of lockdown in the different provinces of Spain.

We will also show the distribution of the infectivity rates of the different provinces of Spain, in an histogram for each stage of the lockdown at Figure 9 and in the Table 4:

Once more, the variance of the distribution of β in the lockdown is very low and we can see how the strict measures have had an effect in the values of β. Although the differences among the infectivity rates from each stage of the de-escalation is less notable at phase 3, we can see a significant contrast between pre-lockdown, lockdown and the phases 0–3.

## 5. Discussion

**Figure 10:**
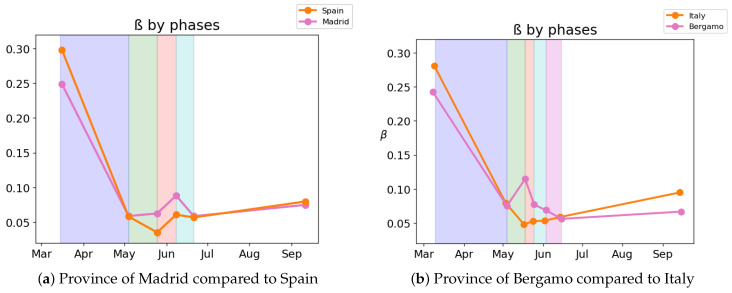
A comparison between the β of the different stages in Spain and Italy.

Although the dependence of the values of the infectivity rate on the different stages is not clear in Figure 2, Figure 4, Figure 6 and Figure 8 it is seen in all the results how the β, which is proportional to the average contact rate between individuals, was generally spiking at the early stages, much higher than during the lockdown, and eventually rose again, when the social distancing measures were relaxed. We observe a clear contrast between the delayed and non-delayed models, in such a way that the *p*-values are lower in the delayed model: the differences in β between each stage are more defined that in the non-delayed. In Spain, significant differences of β are observed between the pre-lockdown and lockdown stages, and between the last phase of lockdown de-escalation and the new normality. From the first easing of the measures to the end of the alarm state period, it can not be concluded that there are evidences that the β has changed. On the other hand, in Italy, the transmission rate varies more significantly during the de-escalating phases. Take note that from Equations (8) and (10), the β is not only dependent on the new cases of infected individuals, but the total number of infectious people, which is a estimation of the total number of infected individuals presented in the population. Thus, the continuous β (blue lines) at the end of summer is not always completely dependent of the new infected individuals (red dotted lines). Observe that in regions such as Abruzzo, Veneto and Calabria in Italy or La Rioja, Asturias in Spain, from Mid-June to Mid-July there are are not many new cases, so the number of total infectious individuals decreases to low levels. Even if it is observed a little growth in new cases, as there is a little number of total infectious individuals, the transmission rate β calculated is high. For this reason, during the periods when there are few cases, the transmission rate is usually more variable and its value can be higher than during the periods with a bigger number of new and total infectious cases. This situation is observed in the mentioned regions. If β is high that does not necessary mean that new cases should be high. We must take into account the number of total infectious there is On the other hand, observe Figure 10a,b where a comparison is made between the infectivity rate β of two provinces and the average of their respective countries. Although Madrid and Bergamo have been specially affected by COVID-19, their β suggest that the initial number of new cases per population was lower than it potentially could be. After the initial lockdown, however, the infectivity rate specially increased in both provinces, suggesting a deficiency or over-relaxation of the measures of social distancing. The contrasts of values of β in the different stages of lockdown is even more pronounced in the case of Spain than in the case of Italy: The spike of the infectivity rate is higher during the pre-lockdown stages, and then back to lesser values later, when the prophylactic measures have been implemented. Observe also in Figure 3, Figure 5, Figure 7 and Figure 9 that during the lockdown, specially in Spain, when the social interaction was equally restricted in all provinces the variance of the values for β is smaller than during the rest of the stages. It is at such times, as the net of social interactions is more prominent, that the diversification of the density of population and the idiosyncrasy of each area is specially poignant.

We can see at Figure 9 that the lockdown was specially effective in Spain, as it reduced the average infectivity rate to half in both models.

## 6. Conclusions

We have seen the impact of the limitations of social interactions and prophylactic measures, such as the use of face masks, in the rate of infection within the population of Italy and Spain during the different stages of spreading of COVID-19.

While there is a clear distinction between the pre-lockdown stages and the lockdown stages, and the “new” normality, authors agree that it is more difficult to establish a contrast between the different intermediate phases, suggesting that the relevant measures affecting the value of β are not taken during the different phases between the high point of the lockdown and the new normality. Also, it suggest that other factors not measured, such as the use of masks and new habits of public hygiene are also important, as it can be seen in the difference of β between pre-lockdown phases and “new normality”.

It can be seen from the histograms from Spain that during the stage of minimal social interaction in the strong lockdown, affecting the whole country in sync, the infectivity rate is not only minimal, but much more homogeneous in all provinces than in any other moment. From this evaluation it can be deduced some kind of average “minimum social interaction”, independent of the idiosyncrasy of the province, probably related to the average number of individuals per household and the sanitary infrastructure of a country, which would be quite homogeneous. Even though the results are not as coherent in Italy as they are in Spain, it is feasible that this is due to the fact that early lockdown was not implemented in all the country at the same time, but gradually as the cases increased to uncontrollable numbers. Also, the limitations and social distancing We hope that new data from other countries such as Portugal, France, Sweden, etc... being currently processed will provide us new insight over this problem. Also, new system of classification regarding the data in which the prophylactic measures implemented in society is advised as, more advanced models such as one with multiple subpopulations, related to their asymptomatic or strong reactions to the disease, or SEIR models in which the exposed subpopulation is taken into account will be also studied in further works.

## Figures and Tables

**Table 1:** Statistics for the Italian Histogram (Non-delayed).

Phase	Mean	Variance	CI 95%	*p*-Value
Pre-lockdown	0.36727	0.03821	(0.33023, 0.40431)
Lockdown	0.07599	0.00004	(0.07482, 0.07716)	0.0
Phase 1	0.05348	0.00059	(0.04889, 0.05807)	0.0
Phase 2	0.08338	0.03196	(0.04951, 0.11726)	0.10092
Phase 3	0.06078	0.00447	(0.04811, 0.07344)	0.23762
Phase 4	0.08749	0.00973	(0.06881, 0.10618)	0.01099
Normality	0.09013	0.00093	(0.08435, 0.09591)	0.26908

**Table 2:** Statistics for the Spanish Histogram (Non-delayed).

Phase	Mean	Variance	CI 95%	*p*-Value
Pre-lockdown	0.22883	0.00078	(0.22111, 0.23655)
Lockdown	0.071	0.00004	(0.06936, 0.07264)	0.0
Phase 0	0.07945	0.01179	(0.04935, 0.10954)	0.92041
Phase 1	0.08555	0.00125	(0.07574, 0.09536)	0.66039
Phase 2	0.07239	0.0013	(0.06239, 0.08239)	0.68447
Phase 3	0.07887	0.00192	(0.06672, 0.09102)	0.71008
Normality	0.11245	0.00291	(0.09749, 0.12741)	0.00067

**Table 3:** Statistics for the Italian Histogram (Delayed).

Phase	Mean	Variance	CI 95%	*p*-Value
Pre-lockdown	0.83841	0.30304	(0.7341, 0.94272)
Lockdown	0.07157	0.00007	(0.06995, 0.0732)	0.0
Phase 1	0.04437	0.00062	(0.03965, 0.04908)	0.0
Phase 2	0.07041	0.00927	(0.05216, 0.08865)	0.00865
Phase 3	0.05583	0.00558	(0.04168, 0.06999)	0.21103
Phase 4	0.07355	0.008	(0.05661, 0.0905)	0.06891
Normality	0.09549	0.00226	(0.08649, 0.1045)	0.00093

**Table 4:** Statistics for the Spanish Histogram (Delayed).

Phase	Mean	Variance	CI 95%	*p*-Value
Pre-lockdown	0.40061	0.00508	(0.38086, 0.42037)
Lockdown	0.06661	0.00004	(0.06477, 0.06844)	0.0
Phase 0	0.06163	0.00401	(0.04407, 0.0792)	0.2545
Phase 1	0.0791	0.00123	(0.06938, 0.08882)	0.11963
Phase 2	0.06847	0.00152	(0.05766, 0.07929)	0.59498
Phase 3	0.07881	0.00315	(0.06326, 0.09436)	0.30296
Normality	0.13141	0.00821	(0.1063, 0.15652)	0.00033

## Data Availability

The authors confirm that the data supporting the findings of this study are available in publicly accessible repositories within the bibliography.

## References

[1] Singh R.K., Rani M., Bhagavathula A.S., Sah R., Rodriguez-Morales A.J., Kalita H., Nanda C., Sharma S., Sharma Y.D., Rabaan A.A. (2020). Prediction of the COVID-19 Pandemic for the Top 15 Affected Countries: Advanced Autoregressive Integrated Moving Average (ARIMA) Model. JMIR Public Health Surveill.

[2] Cotta R.M., NAveira-Cotta C.P., Magal P. (2020). Mathematical Parameters of the COVID-19 Epidemic in Brazil and Evaluation of the Impact of Different Public Health Measures. Biology.

[3] Singh R.K., Drews M., De la Sen M., Kumar M., Singh S.S., Pandey A.K., Srivastava P.K., Dobriyal M., Rani M., Kumari P. (2020). Short -Term Statistical forecasts of COVID-19 infections in India. IEEE Access.

[4] Li M.Y., Graef J.R., Wang L., Karsai J. (1999). Global dynamics of a SEIR model with varying total population size. Math. Biosci..

[5] Nistal R., De la Sen M., Alonso-Quesada S., Ibeas A. (2019). On a New Discrete SEIADR Model with Mixed Controls: Study of Its Properties. Mathematics.

[6] Wang X., Wang Z., Shen H. (2019). Dynamical analysis of a discrete-time SIS epidemic model on complex networks. Appl. Math. Lett..

[7] Koide C., Seno H. (1996). Sex ratio features of two-group SIR model for asymmetric transmission of heterosexual disease. Math. Comput. Model..

[8] De la Sen M., Ibeas A., Alonso-Quesada S., Nistal R. (2019). On a SIR Model in a Patchy Environment Under Constant and Feedback Decentralized Controls with Asymmetric Parameterizations. Symmetry.

[9] Bjørnstad O.N., Finkenstädt B.F., Grenfell B.T. (2002). Dynamics of measles epidemics: Estimating scaling of transmission rates using a time series SIR model. Ecol. Monogr..

[10] Berge T., Lubuma J.S., Moremedi G.M., Morris N., Kondera-Shava R. (2017). A simple mathematical model for ebola in Africa. J. Biol. Dyn..

[11] Shulgin B., Stone L., Agur Z. (1998). Pulse vaccination strategy in the SIR epidemic model. Bull. Math. Biol..

[12] Ambrosio B., Aziz-Alaoui M.A. (2020). On a coupled time-dependent SIR models fiting with New York and New-Jersey states COVID-19 Data. Biology.

[13] McCallum J.H. (2001). Barlow, N. How should pathogen transmission be modeled?. Trends Ecol. Evol..

[14] Rohani P., Keeling M.J. (2008). Modeling Infectious Diseases in Humans and Animals.

[15] D’Onofrio A. (2002). Stability properties of pulse vaccination strategy in SEIR epidemic model. Math. Biosci..

[16] Shend Q., Zhang X., Fan B., Wang C., Zeng B., Li Z., Li X., Li H., Long C., Xuc H. (2020). Diagnosis of the Coronavirus disease (covid-19): RRT-PCR or CT?. Eur. J. Radiol..

[17] Rohrer K., Bajnoczki C., Socha A., Voss M., Nicod M., Ridde V., Koonin J., Rajan D., Koch K. (2020). Governance of the Covid-19 response: A call for more inclusive and transparent decision-making. BMJ Glob. Health.

[18] Barton C.M., Alberti M., Ames D., Atkinson J.A., Bales J., Burke E., Chen M., Diallo S.Y., Earn D.J., Fath B. (2020). Call for transparency of COVID-19 models. Science.

[19] Stokes L.S., Pierce R., Aronoff D.M., McPheeters M.L., Omary R.A., Spalluto L.B., Planz V.B. (2020). Transparency and trust during the Coronavirus disease 2019 (Covid-19) Pandemic. J. Am. Coll. Radiol..

[20] De la Sen M., Ibeas A., Alonso-Quesada S. (2012). On vaccination controls for the SEIR epidemic model. Commun. Nonlinear Sci. Numer. Simul..

[21] Tang B., Scarabel F., Bragazzi N.L., McCarthy Z., Glazer M., Xiao Y., Heffernan J.M., Asgary A., Ogden N.H., Wu J. (2020). De-Escalation by reversing the escalation with a stronger synergistic package of contact tracing, quarantine, isolation and personal protection: Feasibility of preventing a COVID-19 rebound in Ontario, Canada, as case study. Biology.

[22] Roussel L. (1992). La famille en Europe occidentale: Divergences et convergences. Population.

[23] Rhodes A., Ferdinande P., Flaatten H., Guidet B., Metnitz P.G., Moreno R.P. (2012). The variability of critical care bed numbers in Europe. Intensive Care Med..

[24] https://www.ine.es/covid/piramides.htm.

[25] https://www.tuttitalia.it/statistiche/indici-demografici-struttura-popolazione/.

[26] Gandhi R.T., Lynch J.B., Del Rio C. (2020). Mild or moderate Covid-19. N. Engl. J. Med..

[27] Tang B., Wang X., Li Q., Bragazzi N.L., Tang S., Xiao Y., Wu J. (2020). Estimation of the transmission risk of the 2019- Covid and its implication for public health interventions. J. Clin. Med..

[28] Tang B., Bragazzi N.L., Li Q., Tang S., Xiao Y., Wu J. (2020). An updated estimation of the risk of transmission of the novel Coronavirus (2019-nCov). Infect. Dis. Model..

[29] Chen N., Zhou M., Dong X., Qu J., Gong F., Han Y., Qiu Y., Wang J., Liu Y., Wei Y. (2020). Epidemiological and clinical characteristics of 99 cases of 2019 novel coronavirus pneumonia in Wuhan, China: A descriptive study. Lancet.

[30] Lauer S.A., Grantz K.H., Bi Q., Jones F.K., Zheng Q., Meredith H.R., Azman A.S., Reich N.G., Lessler J. (2020). The Incubation Period of Coronavirus Disease 2019 (COVID-19) From Publicly Reported Confirmed Cases: Estimation and Application. Ann Intern Med..

[31] Spanish Database. https://cnecovid.isciii.es/covid19.

[32] Italian Database. https://github.com/pcm-dpc/covid-19.

[33] Bassetti S., Bischoff W.E., Sheretz R.J. (2005). Are SARS superspreaders cloud adults?. Emerg. Inf. Dis..

[34] Lowen A.C., Mubareka S., Steel J., Palese P. (2007). Influenza Virus Transm. Is Depend. Relat. Humidity Temp. PLoS Pathog..

[35] Spain Lockdown Plan. https://www.ecestaticos.com/file/586e3fe4193e6c9f05dca2c2e757b0c7/1588103416-plan-de-transicion-hacia-la-nueva-normalidad.pdf.

[36] https://www.unipd.it/sites/unipd.it/files/2020/DPCM-1-marzo-2020.pdf.

[37] Marcus B.P. (2010). The weighted moving average technique. Wiley Encyclopedia of Operations Research and Management Science.

[38] Italian Official Repository. http://www.salute.gov.it/portale/news/p3_2_1_1_1.jsp?lingua=italiano&menu=notizie&p=dalministero&id=5077.

[39] Montgomery D.C., Runger G.C. (2010). Applied Statistics and Probability for Engineers.

[40] Huang C., Wang Y., Li X., Ren L., Zhao J., Hu Y., Zhang L., Fan G., Xu J., Gu X. (2020). Clinical features of patients infected with 2019 novel coronavirus in Wuhan, China. Lancet.

